# Supplementary material for: Polyketide synthase gene clusters in the ericoid mycorrhizal fungus Oidiodendron maius: genome-wide prediction, expression profiling, and functional characterization of knockout mutants
Source: Front Plant Sci. 2026 Sep 16;17:1924430. doi: 10.3389/fpls.2026.1924430 (PMC13623903; doi:10.3389/fpls.2026.1924430)
Supplement: Supplementary file 1 [file SupplementaryFile1.zip › Supplementary Materials/Supplementary Folder 1.zip!/Oidma1_AssemblyScaffolds_Repeatmasked/knownclusterblast/cluster7/jgi.p_Oidma1_107189_mibig_hits.html]

| MIBiG Protein | Description | MIBiG Cluster | MiBiG Product | % ID | % Coverage | BLAST Score | E-value |
| --- | --- | --- | --- | --- | --- | --- | --- |
| KGO40481.1 | Major\_facilitator\_superfamily\_domain,\_general\_substrate\_transporter | BGC0001205 | Communesin\_biosynthetic\_gene\_cluster | 34 | 92.700729927 | 267 | 4.6e-71 |
| EFY94507.1 | vitamin\_H\_transporter,\_putative | BGC0000337 | Destruxins\_biosynthetic\_gene\_cluster | 32 | 69.3430656934 | 173 | 9e-43 |
